# Coronary Flow in Patients With Myocardial Bridges, Coronary Fistulae in the Setting of Unstable Non-Obstructive Coronary Disease

**DOI:** 10.7759/cureus.13130

**Published:** 2021-02-04

**Authors:** Niya E Semerdzhieva, Stefan Denchev

**Affiliations:** 1 Emergency, National Heart Hospital, Sofia, BGR; 2 Cardiology, Medical Center 'Mediva', Sofia, BGR

**Keywords:** myocardial bridges, coronary fistulae, acute coronary syndrome, non-obstructive coronary disease, slow coronary flow

## Abstract

Оbjective

Our aim was to describe the difference in epicardial coronary flow at baseline on background anti-ischaemic therapy and following intracoronary glyceryl trinitrate in patients with acute coronary syndrome and non-obstructive coronary disease with and without myocardial bridges and coronary artery fistulae.

Materials and methods

Coronary flow was characterized in a group of 88 patients with coronary stenoses <50% diagnosed with acute coronary syndrome using the corrected Thrombolysis in Myocardial Infarction frame count (cTFC) method at coronary angiography at baseline and after the application of 200 µg glyceryl trinitrate.

Results

Тhe patients with myocardial bridges and coronary artery fistulae accounted for 4.4% (n=4) and 2.2% (n=2), respectively, of the patients with acute coronary syndrome. Sixty-two (70%) of all patients demonstrated slow progression of the contrast media (cTFC>25 frames) in at least one coronary artery. Coronary flow was similarly impaired in the patients with myocardial bridges, coronary artery fistulae, and those without coronary anomalies and variants. After the intracoronary infusion of glyceryl trinitrate, the epicardial flow improved in the patients with myocardial bridges and to a lesser degree in the cases with coronary fistulae. Most of the patients who responded to glyceryl trinitrate were on background therapy with calcium channel blockers.

Conclusion

The epicardial coronary flow of patients with non-obstructive coronary disease with myocardial bridges and acute coronary syndrome showed less impairment compared to baseline in response to intracoronary glyceryl trinitrate applied at background anti-ischaemic therapy that included calcium channel blockers.

## Introduction

Myocardial bridges (MBs) are congenital anatomic variants in the normal location of a coronary artery in the epicardium with intramyocardial passage of a coronary segment of different length [1]. Their incidence varies from 0.5-2.5% in angiographic studies, while they are encountered more frequently in autopsy studies (15-85%) [1]. Myocardial bridges may present with symptoms of stable angina, acute coronary syndrome (ACS), coronary spasm, arrhythmia at exercise, heart block, reversible left ventricular dysfunction (myocardial stunning), syncope, or sudden cardiac death [1]. Myocardial bridges are classified based on the severity of symptoms (according to Schwarz) into types A (asymptomatic), B (associated with stress-induced myocardial ischaemia), and C (that alter the haemodynamics of coronary flow) [2]. According to the severity of reduction in the lumen of a coronary artery (Noble's grading), myocardial bridges are classified into grade I, up to 50% narrowing; grade II, >50-75% narrowing; and grade III, >75% narrowing of the epicardial segment lying underneath the myocardial bridges [3]. Quantitative coronary angiography is the main diagnostic method for the assessment of the severity of coronary lumen systolic reduction [1].

A coronary artery fistula (CAF) is an abnormal communication between a coronary artery and another cardiovascular structure that may include a cardiac chamber, a coronary sinus, the superior vena cava, or a pulmonary artery bypassing the capillary bed [4]. The incidence of coronary artery fistulae was 0.2% in all patients undergoing coronary angiography. Fistulous communications may be congenital (representing 0.3% of congenital cardiac disease) or acquired [4,5]. The dimensions of a coronary artery fistula determine the severity of symptoms and the risk of adverse events [4]. Coronary artery fistulae that cause diversion of a significant amount of blood flow and decrease the normal perfusion of myocardial tissue can cause ischaemia (coronary steal phenomenon) and can present as ACS. Rarely, fistulae are associated with coronary embolism and acute myocardial infarction (MI) [4]. A stress perfusion imaging study in patients with coronary artery fistulae shows defects in perfusion subsequent to ischaemia and subendocardial infarcts. The localization of the anatomic focus is achieved by means of coronary computed tomography angiogram and also by cardiac catheterization and coronary angiography [4, 5].

Our aim was to describe the difference in epicardial coronary flow at baseline on background anti-ischaemic therapy and following intracoronary glyceryl trinitrate in patients with acute coronary syndrome and non-obstructive coronary artery disease (NCAD) with and without myocardial bridges and coronary arterial fistulae.

## Materials and methods

In a study group of 88 patients with coronary stenoses <50% (non-obstructive coronary disease) who were admitted for a worsening of the symptoms of angina to University Hospital ‘Alexandrovska’ from June 2006 to March 2008, coronary flow was analysed by coronary angiography.

The diagnoses of ACS, hypertension, dyslipidemia, and diabetes mellitus were made in accordance with the guidelines of the European Society of Cardiology and the European Association for Study of Diabetes. Current smoking was considered as a risk factor. Information about medication usage (angiotensin-converting enzyme inhibitor [ACE-I] or angiotensin receptor blocker [ARB]; beta-blocker; calcium channel blocker; oral nitrate; antithrombotic agent [aspirin or clopidogrel and statin]) was collected. The indications for coronary angiography were non-response to medical treatment of symptoms, electrocardiogram (ECG) with signs of ischaemia at rest or induced by exercise stress test, or an increase in cardiospecific troponin T (cTnT). Angiography was performed with a femoral approach using non-ionic contrast medium (Iopamidol 370) without stopping therapy. At least two orthogonal views for the right coronary artery (RCA) and at least five views (including two orthogonal views) for the left anterior descending coronary artery (LAD) were analysed. The flow in the coronary arteries was assessed using the corrected Thrombolysis in Myocardial Infarction frame count (cTFC) described in previous studies [6]. The TFC measures the number of frames needed for the angiographic media injected into a coronary artery to reach a certain distal landmark. Тhe contrast media was injected simultaneously at the beginning of cardiac systole. The TFC was corrected for the length of the LAD by dividing the TFC measured in the LAD by 1.7. The cTFC before and after the intracoronary application of 200 µg glyceryl trinitrate was measured. The angiograms were recorded at a speed of 12.5 frames/s. To equalize the cTFC results, a second estimation was made so that the coronary flow velocity corresponded to 30 frames/s.

Fifty-three (59%) patients conducted a symptom-limited exercise stress electrocardiographic test on the modified Bruce protocol. Provocation of typical angina, angina-like symptoms, ST depression >2 mm in two electrocardiographically associated precordial leads and/or >1 mm in extremity leads were criteria for positive exercise stress test in the data analysis. Equivocal exercise stress tests were excluded from further analysis.

Left ventricular systolic and diastolic function was assessed by two dimensional-mode echocardiography, Pulsed-Wave Doppler, and tissue Doppler imaging according to standard criteria [7,8]. Left ventricular hypertrophy was defined as interventricular septum or left ventricular posterior wall thickness >12 mm on echocardiography.

Previous coronary revascularization procedures, thrombolytic treatments of myocardial infarction, left ventricular systolic dysfunction, wall motion abnormalities at rest, valve disease, idiopathic cardiomyopathy, acute or chronic inflammatory disease, fractures or wounds, neoplastic disease, suboptimal angiographic imaging, or incomplete data comprised the exclusion criteria for the study.

A history of subsequent hospital readmissions with a diagnosis of acute coronary syndrome (ACS, unstable angina, and acute MI) for a period of 28.7±29.2 months (1-89 months) was obtained by follow-up visits, telephone interviews or medical records of hospital readmissions.

All patients signed written informed consent forms for all diagnostic tests. This retrospective study was approved by the Ethics Committee of University Hospital ‘Alexandrovska’ and complied with the Declaration of Helsinki.

Statistical Package for Social Sciences, version 19, software (IBM Corp., Armonk, NY, USA) was used for the analysis of data. Continuous variables are presented as means and standard deviations (SDs). Categorical variables are presented as counts and percentages. Chi-square tests or Fisher’s exact tests were used for comparisons of the categorial variables. The normality of the continuous variables was analysed with Kolmogorov-Smirnov and Shapiro-Wilk tests. Тhe variables with a normal distribution were compared with Student’s t-test or Welch’s test. The variables without a normal distribution were compared using the Mann-Whitney U test. P-values lower than 0.05 were considered statistically significant.

## Results

Sixty-two patients (70%) demonstrated slow progression of the contrast media (cTFC>25 frames) in at least one coronary artery. One patient (1.1%) was diagnosed with non-ST segment elevation acute myocardial infarction based on symptoms, ECG, and laboratory test results. The rest were patients with unstable angina with NCAD, and their cTnT results remained within the reference range at repeated testing (Table 1).

**Table 1:** Study group - risk profile, echocardiographic and angiographic data NCAD - non-obstructive coronary disease; CAF - coronary artery fistulae; MB - myocardial bridges; DM – diabetes mellitus; LVH – left ventricular hypertrophy; МI – myocardial infarction; Exercise ECG – exercise electrocardiography; CPK - creatinine phosphokinase; CPK-MB - MB fraction of CPK; cTnT – troponin T; Dves  – epicardial lumen diameter; cTFC – corrected TIMI frame count; ACE-I/ARB – inhibitor of angiotensin-converting enzyme/angiotensin receptor blocker; BB – therapy with beta blocker; BB+N – intake of beta blocker and long-acting oral nitrate; BB+CCB – therapy with beta blocker and calcium channel blocker; BB+CCB+N – combination of beta blocker, calcium channel blocker with long-acting nitrate; EDV, ESV – end-diastolic, end-systolic volume; EF – ejection fraction; IVS - interventricular septum; LVPW - left ventricular posterior wall;  E/E’ – peak early diastolic mitral inflow velocity to Doppler-derived mitral annular early diastolic velocity; PASP – CW-Doppler derived systolic pressure in pulmonary artery

Characteristics	NCAD, n (%) n = 82	CAF, n (%) n = 2	МB, n (%) n = 4
Men/Women	27/57 (32/68)	1/1 (50/50)	1/3 (25/75)
Age, years	57.3 ± 8.5	53 ± 5.7	63.8 ± 11.5
Hypertension	70 (84)	2 (100)	4 (100)
DM	13 (15)	0 (0)	1 (25)
Dyslipidemia /75 patients/	58 (76)	2 (100)	2 (50)
Obesity	23 (27)	1 (50)	1 (25)
Smoking	13 (15)	2 (100)	1 (25)
LVH	27 (33)	1 (50)	4 (100)
Prior МI	5 (6)	0 (0)	0 (0)
Positive stress ЕCG /40 patients/	30 (58.8)	1 (50)	2 (50)
CPK, IU/l	117.4 ± 108.3	124.5 ± 55.9	202.3 ± 146.1
CPK-MB, IU/l	15 ± 17.1	16 ± 2.8	12.7 ± 7.1
cTnT, ng/ml	<0.01 ± 0.0	<0.01 ± 0.0	<0.01 ± 0.0
Dves, mm	3.6 ± 0.8	3.4 ± 0.1	3.3 ± 0.5
сTFC, frames	32.7 ± 13.3	32.5 ± 3.5	33 ± 16.7
Atherosclerotic plaques	30 (35)	-	2 (50)
ACE-I/АRB	56 (67)	2 (100)	3 (75)
Statin	52 (67)	2 (100)	3 (75)
Aspirin/Clopidogrel	69 (84)	2 (100)	3 (75)
No anti-ischaemic therapy	8 (10)	0 (0)	0 (0)
BB	33 (43)	1 (50)	0 (0)
BB+N	16 (21)	0 (0)	1 (25)
BB+CCB	14 (18)	1 (50)	2 (50)
BB+CCB+N	31 (25.6)	0 (0)	1 (25)
EDV, ml	115.3 ± 23.2	89 ± 18.7	94.3 ± 18.2
ESV, ml	37.6 ± 12	23.5 ± 4.4	27.8 ± 7.2
EF, %	67.4 ± 6.3	72.3 ± 2.1	70 ± 4.2
IVS/LVPW, mm	11.3±1.6/11.3±1.4	11±1.4/11.8±0.4	13.9±1.3/13.6±1.1
Left ventricular filling types			
-impaired relaxation	10 (25.6)	0 (0)	-
-pseudonormal	5 (12.8)	2 (100)	2 (50)
-restrictive	2 (5.1)	0 (0)	-
E/E'	9 ± 2.8	10.5± 2.1	9.5± 3.5
PASP, mmHg	29±5	25±1.4	28.5±5

Myocardial bridges and coronary artery fistulae were encountered in 4.4% and 2.2%, respectively, of the patients with unstable non-obstructive coronary disease. The patients with myocardial bridges were older than the patients with coronary artery fistulae. In all cases the myocardial bridge was located in a segment of LAD and in all but one patient they caused insignificant reduction of the coronary lumen in systole.

Following the intracoronary application of glyceryl trinitrate in LAD, the epicardial coronary flow showed improved characteristics in those with myocardial bridges and to a certain degree in the subset of those with coronary artery fistulae (Tables 2). There was an insignificant difference in the degree of flow impairment between coronary artery fistulae, myocardial bridges, and NCAD at baseline (Table 3). The postnitrate coronary flow of the patient group with myocardial bridges was less disturbed compared with that of the rest NCAD patients (Table 3).

**Table 2:** Effects of intracoronary glyceryl trinitrate on epicardial coronary flow NACD - non-obstructive coronary disease; Dves, Dvesn - epicardial coronary diameter before and following glyceryl trinitrate; cTFC, cTFCn - baseline and postnitrate corrected Thrombolysis in Myocardial Infarction frame count; CAF - coronary artery fistulae; MB - myocardial bridges; GTN - glyceryl trinitrate; NS - not significant statistically

Patient group/variable	Before GTN	After GTN	Δ	p-value
NCAD				
Dves	3.6 ± 0.8	3.9 ± 0.8	- 0.3 ± 0.4	<0.0001
cTFC	32.4 ± 11.7	33.1 ± 11.6	-0.7 ± 10.7	0.554
CAF				
Dves	3.4 ± 0.1	3.7 ± 0.3	-0.3 ± 0.4	NS
cTFC	32.5 ± 3.6	31.5 ± 14.8	1 ± 11.3	NS
MB				
Dves	3.3 ± 0.5	3.7 ± 0.4	-0.3 ± 0.2	NS
cTFC	33 ± 16.7	27.6 ±12.2	5.4 ± 5.7	NS

**Table 3:** Epicardial flow at baseline and after intracoronary GTN, a comparison of patients with non-obstructive coronary disease, coronary fistulae and myocardial bridges NCAD - non-obstructive coronary disease; CAF - coronary artery fistulae; MB - myocardial bridges; Dves, Dvesn– epicardial lumen diameter before and after glyceryl trinitrate; cTFC, cTFCn – baseline and post-nitrate corrected Thrombolysis in Myocardial Infarction frame count; GTN - glyceryl trinitrate; NS - not significant statistically

Variable/patient group	N	NCAD	CAF	p-value
Dves	82/2	3.6 ± 0.6	3.4 ± 0.1	NS
cTFC	82/2	32.7 ± 11.6	32.5 ± 3.5	NS
Dvesn	82/2	3.9 ± 0.8	3.7 ± 0.3	NS
cTFCn	82/2	33.1 ± 11.6	31.5 ± 14.8	NS
		NCAD	МB	p-value
Dves	82/4	3.6 ± 0.8	3.3 ± 0.5	NS
cTFC	82/4	32.4 ± 11.6	33 ± 16.7	NS
Dvesn	82/4	3.9 ± 0.8	3.7 ± 0.4	NS
cTFCn	82/4	33.1 ± 11.6	27.6 ± 12.1	NS

The majority of patients with myocardial bridges and coronary artery fistulae were on background therapy with beta-blockers plus calcium channel blockers with or without oral nitrate, in contrast to those with NCAD. Тhe intake of calcium channel blockers was associated with less impaired coronary flow than therapy with beta-blockers as the sole anti-ischaemic medication and beta-blockers plus nitrates (Table 4).

**Table 4:** Intake of calcium channel blockers - relation to coronary flow in patients with NCAD, myocardial bridges, and coronary artery fistulae NCAD - non-obstructive coronary disease; CAF - coronary artery fistulae; MB - myocardial bridges; Dves, Dvesn - baseline and epicardial coronary artery diameter; cTFC, cTFCn - corrected Thrombolysis in Myocardial Infarction frame counts before and after glyceryl trinitrate; BB/BB+N - therapy with beta-blocker or beta-blocker plus long-acting nitrate; BB+CCB±N - combination of beta-blocker, calcium channel blocker with long-acting nitrate; NS - not significant statistically

Patient group/variable	N	BB/BB+N	BB+CCB±N	p-value
NCAD				
Dves	45/19	3.5 ± 0.7	3.5 ± 0.6	0.944
cTFC	45/19	32.1 ± 12.1	27.1 ± 7.7	0.056
Dvesn	45/19	3.8 ± 0.6	3.8 ± 0.8	0.825
cTFcn	45/19	32.5 ± 10.3	27.9 ± 9.5	0.115
CAF				
Dves	1/1	3.3	3.5	NS
cTFC	1/1	35	30	NS
Dvesn	1/1	3.9	3.5	NS
cTFCn	1/1	42	21	NS
MB				
Dves	1/3	3.8	3.2 ± 0.5	-
cTFC	1/3	46	29.3 ± 16.6	-
Dvesn	1/3	4.3	3.6 ± 0.4	-
cTFCn	1/3	31	25 ± 12.4	

## Discussion

The results of the distribution of myocardial bridges in patients admitted with a diagnosis of ACS are similar to those of Rossi et al., who demonstrated a 2.1% incidence of myocardial bridges in a population with acute coronary disease [9]. In our study, there were two cases of coronary artery fistulae that represented small distal communications of the LAD. One of them was between the distal LAD and the left ventricle, and the other was a communication of the LAD with the coronary vein. In both of the patients, no signs of cardiac overload were detected, either at the physical examination or at echocardiography. Coronary artery fistulae were relatively common in our study population. A possible explanation is that they are more frequently encountered in subpopulations with acute coronary disease compared to the distribution among patients with diverse cardiovascular pathologies undergoing cardiac catheterization and coronary angiography. The incidence of coronary artery fistulae was 0.5% (6/1,190 patients) in a subgroup with ST-segment elevation myocardial infarction according to Chui et al. [10].

We found similarly disturbed coronary flow in the patients with myocardial bridges, coronary artery fistulae and the rest of our study group with NCAD. Earlier studies using the method cTFC in patients with myocardial bridges have shown various degrees of disturbance in epicardial flow compared to control individuals without coronary disease [11, 12]. Prior data suggested that dysfunction in the microvascular circulation is a common pathologic mechanism of ischaemia that operates in addition to specific mechanisms in patients with myocardial bridges [12-14]. This may be due to a limited hyperemic response as a result of delayed relaxation in diastole of the bridge segment [14]. Coronary spasm has also been described in coronary artery fistulae as a contributing factor to delayed epicardial flow [15, 16].

In our study, the application of glyceryl trinitrate led to significant acceleration [17] in coronary flow in the cases of NCAD with myocardial bridges and produced almost no effect on the flow in the subgroup with coronary artery fistulae and the rest of the study population. The observed effect of glyceryl trinitrate in our patients is opposite to what has been found in earlier studies of patients with long myocardial bridges with great muscular index. It has been demonstrated that the alterations in heart rate and systemic arterial pressure induced by the intracoronary application of glyceryl trinitrate in patients receiving no other medication caused augmentation of the pressure gradient in the coronary segment with the intramyocardial passage [18]. In our opinion, the observed effect on flow exerted by glyceryl trinitrate is related to haemodynamically insignificant coronary abnormalities (superficial myocardial bridges and fewer myocardial fibres) and was in part related to the favourable impact of a therapeutic combination that includes beta-blockers and calcium channel blockers (selective arteriolar vasodilators) regarding the coexisting microvascular dysfunction and the impaired left ventricular diastolic function [19-21]. All patients with coronary anomalies and variants in our study were on therapy with a beta-blocker. Additional improvement of flow was observed in those patients taking combined therapy that included a calcium channel blocker.

Myocardial bridges could accelerate the progression of coronary disease [1, 22, 23]. The clinical course of coronary artery fistulae is highly variable, but the majority of fistulae enlarge over time and eventually become symptomatic [4, 24]. Grade III and type C myocardial bridges, despite maximal anti-ischaemic therapy, should undergo minimally invasive coronary bypass and myocyte lysis or cardiopulmonary bypass (for myocardial bridges >2.5 сm in length, located 5 mm and deeper in the myocardium) [25]. Specific management strategies, which can include surgical repair, percutaneous catheter embolization or surgical closure for the treatment of symptomatic coronary artery fistulae, have been controversial because of the high incidence (11%) of postoperative myocardial infarction and reduced late survival compared with an age-matched population [5]. Our patients with myocardial bridges and coronary artery fistulae received only conservative treatment because of mild symptoms and lack of obstructive coronary disease. As a result, they were not referred for further imaging studies. They were also advised to seek diagnostic evaluation of the contribution of non-cardiac comorbidities to symptoms because this could be useful in building treatment strategies [4]. Only about half of the study group was re-evaluated in the follow-up period. Among these was one patient with coronary variant (diagnosed with a myocardial bridge) and he reported neither deterioration of symptoms nor ischaemic-driven hospital readmissions.

## Conclusions

In a study group with unstable non-obstructive coronary disease and high prevalence of slow coronary flow at rest, the epicardial coronary flow of the patients with myocardial bridges showed less impairment compared to baseline in response to intracoronary glyceryl trinitrate, and this response can be related to background anti-ischaemic therapy that included calcium channel blockers.
